# Supplementary material for: SARS-CoV-2 Serostatus and COVID-19 Illness Characteristics by Variant Time Period in Non-Hospitalized Children and Adolescents
Source: Children (Basel). 2023 Apr 30;10(5):818. doi: 10.3390/children10050818 (PMC10217706; doi:10.3390/children10050818)
Supplement: Supplementary file 1 [file children-10-00818-s001.zip › children-2291614-supplementary.pdf]

Supplementary Materials

Table S1. Vaccination Status Across Four SARS-CoV-2 Variant Time Periods. .

Vaccination Status	Variant Time Period			
	**Pre-Delta N=1701**	**Delta N=3309**	**Omicron N=1635**	**Omicron BA.2 N=1316**
**Full n (%)**	20 (1.18%)	1038 (31.37%)	908 (55.54%)	787 (59.8%)
**Partial n (%)**	18 (1.06%)	196 (5.92%)	48 (2.94%)	17 (1.29%)
**None n (%)**	1663 (97.77%)	2075 (62.71%)	679 (41.53%)	512 (38.91%)

Table S2. Symptom Severity by Variant Time Period.

Symptom Severity	Variant Time Period			
	**Pre-Delta N=1701**	**Delta N=3309**	**Omicron N=1635**	**Omicron BA.2 N=1316**
**Mild n (%)**	44 (2.59%)	240 (7.25%)	414 (25.32%)	516 (39.21%)
**Severe n (%)**	(0%)	5 (0.15%)	9 (0.55%)	6 (0.46%)
**Missing n (%)**	1657 (97.41%)	3064 (92.6%)	1212 (74.13%)	794 (60.33%)
